# Relationships among deliberate practice, task persistence, perceived challenge, and creative performance in physical education classes of Chinese secondary school students: a cross-sectional study

**DOI:** 10.3389/fpsyg.2026.1741430

**Published:** 2026-03-19

**Authors:** Jilin Li, Xiaohui Jiang, Xuehui Zhang

**Affiliations:** 1 Sports Institute of Hefei Normal University, Hefei, Anhui, China; 2 School of Physical Education, Jinggangshan University, Ji'an, China

**Keywords:** creative performance, deliberate practice, perceived challenge, secondary school students, task persistence

## Abstract

**Background:**

Creativity in physical education (PE) has become increasingly important in educational psychology, particularly in China, where traditional teaching methods are being combined with more student-centered and cognitively engaging practices. However, research on the influence of motivational and cognitive factors, such as deliberate practice, task persistence, and perceived challenge, on creative performance among secondary school students in PE settings.

**Objective:**

This study aimed to explore the interrelationships among deliberate practice, task persistence, perceived challenge, and creative performance in physical education classes of Chinese secondary school students.

**Methods:**

A cross-sectional survey was conducted with 823 students aged 13 to 17 from six secondary schools in Anhui Province, China. Validated standardized instruments were used to measure deliberate practice, task persistence, perceived challenge, and creative performance. Structural equation modeling (SEM) was employed to test the hypothesized relationships among these variables and assess any mediating effects.

**Results:**

The analysis revealed that deliberate practice had a significant positive effect on creative performance (*β* = 0.309, *p* < 0.01), both directly and indirectly through task persistence and perceived challenge. Task persistence partially mediated the relationship between deliberate practice and creativity (effect = 0.138, 0.065–0.219). Perceived challenge served as a full mediator between task persistence and creative outcomes (effect = 0.085, 0.045–0.135). Additionally, task persistence and perceived challenge jointly mediated the relationship between deliberate practice and creative outcomes (effect = 0.045, 0.021–0.076). The final SEM model demonstrated good fit indices, providing support for the hypothesized framework.

**Conclusion:**

The findings highlight the significance of structured effort, persistence, and optimal cognitive challenge in promoting creative performance in PE. By incorporating strategies for deliberate practice and encouraging sustained engagement with challenging tasks, it is possible to enhance creativity among Chinese adolescents in physical education settings.

## 1 Introduction

Creativity in physical education (PE) is becoming an increasingly important focus within educational psychology and pedagogy, particularly in China, where traditional teaching methods are being enhanced with learner-centered and cognitively engaging approaches (Cremin and Chappell, 2019). Creative performance in PE not only reflects the acquisition of physical skills but also showcases an individual’s ability to adapt, solve problems, and express themselves innovatively through movement. Therefore, understanding the cognitive and motivational factors that contribute to creativity has become essential.

Deliberate practice, defined as structured, effortful, and goal-oriented activity aimed at enhancing performance (Anders Ericsson et al., 2007), has been extensively studied in the context of talent development. While previous research confirms its role in improving motor and cognitive performance (Macnamara et al., 2016), the focus has primarily been on performance efficiency rather than creative outcomes. Specifically, there has been limited exploration of how deliberate practice may foster creativity in everyday educational settings, such as school PE. Thus, while its effectiveness for skill acquisition is well established, its potential role in promoting creative performance remains underexplored.

Task persistence, which closely aligns with the concept of grit, refers to sustained effort toward a goal despite facing challenges (Duckworth et al., 2010). Persistent engagement in tasks can influence how students navigate difficulties and maintain motivation. However, persistence alone does not necessarily guarantee creativity; its effectiveness may depend on how students interpret and respond to the demands of the task. This suggests that additional psychological mechanisms may clarify how persistence translates into creative performance.

Perceived challenge, defined as a student’s subjective assessment of the difficulty of a task in relation to their abilities (Teuber et al., 2021), may represent such a mechanism. When challenges are perceived as attainable rather than overwhelming, they can enhance intrinsic motivation and facilitate deeper learning (Liu et al., 2025). Conversely, a mismatch between challenge levels and student abilities may hinder engagement and restrict creative exploration. Although both deliberate practice and task persistence involve sustained effort, there has been little research examining whether perceived challenge acts as a linking mechanism that connects these effort-based behaviors to creative outcomes in PE settings.

Existing literature has largely explored deliberate practice, persistence, and perceived challenge in isolation. Few studies have integrated these variables into a comprehensive explanatory framework within physical education, and even fewer have investigated their sequential relationships. Therefore, the originality of this study lies not only in its geographical context but also in its integrative modeling approach. By proposing a chain mediation model, this study moves beyond examining direct associations to investigate how deliberate practice may influence creative performance through task persistence and perceived challenge as sequential psychological processes.

Moreover, while Chinese secondary school students operate within a cultural environment that emphasizes effort, perseverance, and academic achievement (Li, 2010), previous research has not sufficiently explored how these culturally reinforced norms regarding effort may interact with motivational mechanisms to shape creativity in PE. Investigating these relationships in this context offers an opportunity to test whether effort-related constructs function differently within a system that strongly values discipline and sustained practice.

To address these gaps, this study explores the relationships among deliberate practice, task persistence, perceived challenge, and creative performance among secondary school students in China’s PE classes. Specifically, it tests a chain mediation model to determine whether perceived challenge serves as a psychological mechanism linking practice-related variables to creative performance. By clarifying these internal motivational pathways, this research contributes to a deeper understanding of how structured effort translates into creative expression within physical education settings.

## 2 Theoretical basis and hypothesis

### 2.1 The relationship between deliberate practice and creative performance

Deliberate practice was initially conceived as a structured and intentional process designed to improve performance through feedback and sustained effort (Ericsson et al., 1993). It is widely recognized not only for its role in skill acquisition but also for its potential to elevate higher-order cognitive and creative capabilities. Unlike repetitive or passive activities, deliberate practice demands active engagement, self-monitoring, and the adjustment of challenges, all of which create favorable conditions for creativity. In physical education contexts, such practice enables students to automate fundamental motor skills, freeing cognitive resources for exploration, improvisation, and innovation (Macnamara et al., 2016; Ericsson and Ward, 2007). This approach is beneficial in scenarios that require flexible adaptation to dynamic movement challenges, a hallmark of creative motor performance (Ueda et al., 2023).

Existing research reveals that creativity in physical tasks is not merely an inherent trait but emerges from accumulated, reflective interactions with task-specific constraints. For example, emphasized that creativity often results from advanced performance capabilities developed through sustained, high-quality practice. Additionally, the expert performance framework illustrates how domain-specific innovation builds upon foundational competence and automaticity, which enables individuals to recombine learned skills in novel and effective ways (Ericsson and Moxley, 2012). In educational settings, particularly in physical education, pedagogical models that integrate elements of deliberate practice—such as goal clarity, targeted feedback, and increasing task complexity—have been associated with improvements in both skill mastery and students’ originality in task execution (Ellison and Woods, 2016; Memmert and Roth, 2007).

Furthermore, research in culturally structured settings suggests that deliberate practice can act as a transformative tool. In the Chinese educational system, which has traditionally prioritized compliance over autonomy, incorporating principles of deliberate practice could shift the focus from mere compliance to a more autonomy-supportive learning environment. This transition would help students not only to gain competence but also to cultivate a mindset oriented toward flexibility and self-initiated performance, which are crucial elements for achieving creative outcomes (Chen et al., 2022). Thus, deliberate practice serves not only as a training method but also as a pedagogical strategy that fosters students’ cognitive and motivational readiness to innovate in physical learning tasks.

In summary, the growing body of evidence indicates that deliberate practice extends beyond technical enhancement, functioning as a catalyst for adaptive and original behaviors within complex physical environments. By integrating deliberate practice into physical education pedagogy, educators can significantly improve students’ opportunities for both skill development and creative expression. Therefore, the following hypothesis is proposed:

*H1*: Deliberate practice is positively associated with creative performance among Chinese secondary school students in physical education classes.

### 2.2 The mediating role of task persistence

Task persistence, often defined as the sustained effort toward long-term goals despite obstacles or setbacks, has garnered increasing attention as a critical psychological factor in educational and performance contexts (Duckworth et al., 2007). In PE, task persistence reflects students’ willingness to consistently engage in skill refinement, problem-solving, and coping with failure—all of which are necessary for developing creative performance (Orth et al., 2018).

Deliberate practice, characterized as structured, goal-directed, and repetitive activities with immediate feedback (Ericsson et al., 1993), is believed to foster persistence by emphasizing incremental mastery and fostering self-regulation. Students who regularly engage in deliberate practice are more likely to develop a higher tolerance for setbacks and cultivate adaptive learning behaviors, enabling them to sustain effort over time (Macnamara and Hambrick, 2021). Consequently, this persistence supports deeper engagement with motor tasks, facilitating creative exploration and flexible problem-solving in physically and cognitively demanding contexts (Evans et al., 2021; Nijstad et al., 2010).

Moreover, persistence is proposed to function as a motivational conduit that transforms the effects of externally structured practice into internalized engagement and innovation (Ryan and Deci, 2000). In PE settings, persistent students tend to remain engaged even while facing complex tasks, thereby increasing their opportunities for trial, error, and creative adaptation. This behavioral tendency aligns with the “challenge engagement hypothesis,” which asserts that sustained effort leads to deeper engagement with moderately difficult tasks, ultimately fostering creativity (Guadagnoli and Lee, 2004; Simón-Chico et al., 2023).

Building on this theoretical framework, the current study posits that task persistence mediates the relationship between deliberate practice and creative performance. Specifically, it was hypothesized that students who engage more frequently and deliberately in practice are more likely to develop greater task persistence, which, in turn, enhances their creative performance in PE settings. This mediating mechanism underscores the motivational pathway through which structured practice contributes to creative outcomes by promoting long-term engagement and resilience in the face of challenges.

*H2*: Task persistence mediates the relationship between deliberate practice and creative performance in Chinese secondary school physical education classes.

### 2.3 The mediating role of perceived challenge

In PE, perceived challenge serves as a crucial motivational factor that transforms repetitive practice into cognitively engaging and adaptive learning experiences (Abós et al., 2021). Closely aligned with flow theory, perceived challenge facilitates optimal engagement when individuals believe their skill level matches the demands of the task (Csikszentmihalyi and Csikzentmihaly, 1990). When students view a task as appropriately challenging, they are more likely to exhibit deep involvement, persistence, and exploratory behavior—conditions under which creativity is most likely to emerge (Huhtiniemi et al., 2021). Recent research in sports pedagogy recognizes perceived challenge as a cognitive-affective bridge linking effortful practice with creative outcomes. Students who report a higher perceived challenge in PE tasks tend to be more intrinsically motivated, showing increased engagement that fosters divergent thinking and flexible problem-solving (Leung et al., 2014). This suggests that the impact of deliberate practice depends not only on the quantity or structure of tasks but also on how students interpret those experiences as opportunities for growth.

In this context, perceived challenge catalyzes the transition from technical rehearsal to adaptive output. For example, Liu and Chang (2023) found that supportive yet appropriately challenging environments in PE significantly enhanced students’ creative strategies and behaviors. This relationship is particularly significant in Chinese educational settings, where traditional instruction often focuses on conformity and performance accuracy. By fostering a climate where students perceive PE tasks as meaningful and stimulating, educators can activate vital motivational resources for creativity, including persistence, emotional investment, and strategic flexibility (Su and Liu, 2025; Miao et al., 2024). Collectively, these findings suggest that perceived challenge acts as a key mediating mechanism that transforms structured, effortful practice into creative action. Therefore, this study proposes the following hypothesis:

*H3*: Perceived challenge mediates the relationship between deliberate practice and creative performance in Chinese secondary school physical education classes.

### 2.4 Construction of the sequential mediator model

Within the framework of skill acquisition and motivation theories, particularly the Expert Performance Framework (Ericsson and Ward, 2007) and Flow Theory (Csikszentmihalyi and Csikzentmihaly, 1990), creative performance in PE is conceptualized as the product of both structured practice and adaptive psychological engagement. When students engage in high levels of deliberate practice, they are more likely to sustain task persistence, reflecting continued effort and resilience despite challenges (Duckworth et al., 2007). This persistent effort enhances students’ perception of challenging tasks as attainable and worthwhile—termed perceived challenge (Teuber et al., 2021).

Research indicates that persistence plays a foundational role in shaping one’s cognitive appraisal of task difficulty. Persistent individuals are more inclined to perceive high task demands as motivating rather than threatening, thereby increasing the likelihood of deep task engagement (Zhang et al., 2025). As a proximal cognitive-affective factor, perceived challenge has been shown to stimulate creative problem-solving and flexible thinking in physical tasks by activating intrinsic motivation and facilitating flow states (Huhtiniemi et al., 2021). Thus, the sequence from deliberate practice to task persistence and then to perceived challenge fosters optimal conditions for creative performance.

This chained mediation model is particularly relevant in Chinese secondary school contexts, where effort-based pedagogy and structured PE practices prevail. Students who persist through deliberate practice are well-positioned to perceive difficult tasks as achievable, thereby promoting motivation-driven creative actions. Therefore, this study proposes the following sequential mediation hypothesis:

*H4*: Task persistence and perceived challenge function as serial mediators in the relationship between deliberate practice and creative performance in Chinese secondary school physical education classes.

**Figure 1:**
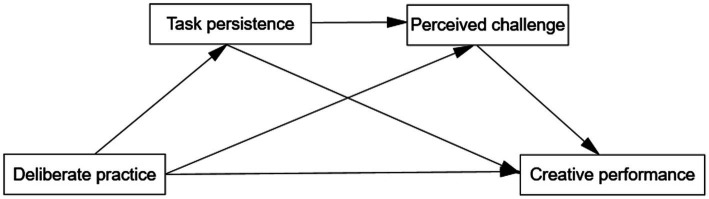
Hypothetical model.

In summary, this study aims to examine the effect of deliberate practice on creative performance in Chinese secondary school PE classes by constructing a sequential mediation model, as illustrated in Figure 1. The study will investigate the following aspects:

(1) Deliberate practice significantly and positively predicts creative performance.
(2) Task persistence and perceived challenge serve as distinct mediators in the relationship between deliberate practice and creative performance.
(3) Task persistence and perceived challenge function as a sequential mediating mechanism between deliberate practice and creative performance in Chinese secondary school physical education classes.
(4) Task persistence and perceived challenge play a mediating role in the sequential relationship between deliberate practice and creative performance.

## 3 Research participants and methods

### 3.1 Procedure and participants

This study was conducted between July and September 2024, focusing on middle and high school students in China. A convenience sampling method was used to recruit participants.

Before the main survey, a pilot study was carried out to evaluate the clarity, reliability, and construct validity of the measurement instruments. A total of 330 questionnaires were distributed to students from middle and high schools in Anhui and Jiangsu provinces. After excluding 8 invalid responses, 322 valid questionnaires were retained for analysis, resulting in a response rate of 98%. The pilot data were analyzed to assess internal consistency and preliminary construct validity. All scales demonstrated satisfactory reliability, with Cronbach’s *α* coefficients exceeding the commonly accepted threshold of 0.70. Exploratory factor analysis showed that item loadings aligned well with the intended factor structure. Based on the pilot results, minor wording adjustments were made to enhance clarity, while no substantial modifications to the scale structure were necessary. The pilot sample was not included in the final dataset for hypothesis testing.

In the main survey, 600 students from middle and high schools across China were selected as the research sample. After removing 65 invalid responses (e.g., incomplete responses or patterned responses), 535 valid questionnaires were retained for final data analysis, resulting in a valid response rate of 89%. Among the participants, 250 were male (45.7%), and 285 were female (53.3%), with 243 being middle school students (45.4%) and 292 being high school students (54.6%).

This study was approved by the Ethics Committee of Hefei Normal University, and all research procedures adhered to the ethical principles outlined in the Declaration of Helsinki.

### 3.2 Research tools

#### 3.2.1 Deliberate practice

The scale for measuring deliberate practice was originally developed by Vallerand et al. (2008) and was later utilized by Cho (2020) and Sim et al. (2024). This unidimensional scale consists of six items with responses measured on a 5-point Likert scale ranging from 1 (“Strongly disagree”) to 5 (“Strongly agree”). The internal consistency of the scale was deemed acceptable, with a Cronbach’s alpha coefficient of 0.805. Confirmatory factor analysis (CFA) results indicated a good model fit: *χ^2^/df* = 2.630, NFI = 0.952, GFI = 0.974, RMSEA = 0.052, CFI = 0.962, and TLI = 0.936.

#### 3.2.2 Task persistence

The Persistence and Effort Scale originally developed by Elliot and Church (1997) and Wentzel (1996), and revised by Guan et al. (2006), was used to measure task persistence in this study. Specifically, the persistence subscale was adopted, consisting of four items. Participants responded on a 5-point Likert scale ranging from 1 (“not at all true”) to 5 (“very true”). The scale demonstrated good internal consistency, with a Cronbach’s alpha coefficient of 0.829. Results from confirmatory factor analysis (CFA) indicated a good model fit: *χ^2^/df* = 2.781, NFI = 0.973, GFI = 0.990, RMSEA = 0.045, CFI = 0.977, and TLI = 0.931.

#### 3.2.3 Perceived challenge

The challenge subscale used in this study was derived from the Student Perceptions of Classroom Quality (SPOCQ) questionnaire developed by Gentry and Owen (2004). This subscale consists of seven items and employs a 5-point Likert scale ranging from 1 (“strongly disagree”) to 5 (“strongly agree”). The reliability and validity of the scale were confirmed, yielding a Cronbach’s alpha coefficient of 0.889, indicating good internal consistency. CFA results demonstrated acceptable model fit: χ^2^/df = 2.660, TLI = 0.942, IFI = 0.967, CFI = 0.937, NFI = 0.925, and RMSEA = 0.035.

#### 3.2.4 Creative performance

The Creative Performance subscale of the *Creative* Self-Efficacy scale, developed by Ha and Cho (2016), was adapted and revised to fit the context of PE classes. The modified scale consists of 11 items and functions as a unidimensional factor. Participant responses were measured using a 5-point Likert scale ranging from 1 (“strongly disagree”) to 5 (“strongly agree”). The internal consistency of this scale was high, with a Cronbach’s alpha of 0.889. CFA results indicated acceptable model fit: χ^2^/df = 2.213, TLI = 0.920, IFI = 0.936, CFI = 0.936, NFI = 0.918, and RMSEA = 0.068.

### 3.3 Statistical analyses

Following data collection, the dataset was imported into SPSS 27.0 for analysis. Initially, incomplete, invalid, and missing questionnaires were removed. Next, the internal consistency reliability of the measurement instruments was assessed. Common method bias was evaluated to ensure the validity of the data. Demographic differences in the main variables were analyzed using independent samples t-tests and one-way ANOVA. Pearson correlation analysis was conducted to explore the relationships among the key variables. Subsequently, AMOS 24.0 was used to perform CFA on each variable and evaluate the model fit of the chain mediation. The influence path and chain mediation effect between variables were tested.

## 4 Results

### 4.1 Common method bias test

To assess the potential impact of common method bias, Harman’s single-factor test was conducted using exploratory factor analysis without rotation, as recommended in prior literature. The analysis included all measurement items from the four study variables: deliberate practice, task persistence, perceived challenge, and creative performance. Four distinct factors were extracted, and the first factor accounted for 32.50% of the total variance—well below the commonly accepted threshold of 40%. These findings suggest that common method variance is unlikely to pose a significant threat to the validity of the results (Podsakoff et al., 2003).

### 4.2 Descriptive statistics and correlation analysis of the variables

This study utilized independent samples t-tests and one-way ANOVA to investigate group differences in deliberate practice (DP), task persistence (TP), perceived challenge (PC), and creative performance (CP) based on gender, educational stage, and sports skill level.

As illustrated in Table 1, no significant gender differences were found in DP, TP, PC, or CP (all *p* > 0.05), indicating that male and female students display similar levels across these key study variables. Likewise, no significant differences were found between middle school and high school students for any of the variables (all *p* > 0.05), suggesting that educational stage does not notably influence students’ engagement with deliberate practice, persistence, challenge perception, or creativity in PE.

**Table 1:** Testing of demographic differences among variables.

Demographic information		Number	DP	TP	PC	CP
Gender	Male	250	3.485 ± 0.771	3.445 ± 0.818	3.276 ± 0.754	3.472 ± 0.695
	Female	285	3.407 ± 0.741	3.449 ± 0.715	3.161 ± 0.684	3.352 ± 0.735
	*t*		1.196	−0.062	1.852	1.933
School level	Middle school	243	3.389 ± 0.779	3.453 ± 0.765	3.220 ± 0.699	3.384 ± 0.699
	High school	292	3.488 ± 0.734	3.441 ± 0.765	3.210 ± 0.737	3.429 ± 0.735
	*t*		−1.502	0.179	0.164	−0.728
Physical skill level	High	154	3.465 ± 0.815	3.508 ± 0.870	3.269 ± 0.797	3.514 ± 0.762
	Medium	290	3.484 ± 0.695	3.505 ± 0.676	3.247 ± 0.628	3.427 ± 0.663
	Low	91	3.276 ± 0.819	3.159 ± 0.780	3.022 ± 0.825	3.177 ± 0.767
	*F*		2.727	7.971^***^	4.047^**^	6.927^***^

*N* = 535; DP, deliberate practice; TP, task persistence; PC, perceived challenge; CP, creative performance. ^**^p < 0.01, ^***^p < 0.001.

In contrast, significant differences emerged based on students’ self-reported physical skill levels. One-way ANOVA revealed that students with high and medium physical skill levels reported significantly greater task persistence (*F* = 7.971, *p* < 0.001), perceived challenge (*F* = 4.047, *p* < 0.01), and creative performance (*F* = 6.927, *p* < 0.001) compared to those with low skill levels. The differences in deliberate practice approached statistical significance (*F* = 2.727, *p* = 0.066), hinting at a trend where students with higher physical skills may engage more in structured practice activities.

Table 2 shows that deliberate practice, task persistence, perceived challenge, and creative performance were all significantly and positively correlated (*p* < 0.01). Specifically, deliberate practice was strongly related to task persistence (*r* = 0.636), perceived challenge (*r* = 0.543), and creative performance (*r* = 0.590). This indicates that students who frequently engage in intentional and structured practice in PE classes tend to persist longer in tasks, perceive greater levels of challenge, and demonstrate higher levels of creative performance. Additionally, task persistence was significantly associated with perceived challenge (*r* = 0.521) and creative performance (*r* = 0.555), suggesting that sustained engagement in tasks positively contributes to students’ experiences of challenge and creativity. The significant positive correlation between perceived challenge and creative performance (r = 0.536) further emphasizes the crucial role of challenge perception in facilitating creativity.

**Table 2:** Means, standard deviations, and correlations among variables.

Variable	M	SD	1	2	3	4
			*r*			
1. DP	3.448	0.749	1			
2. TP	3.447	0.764	0.636^**^	1		
3. PC	3.215	0.719	0.543^**^	0.521^**^	1	
4. CP	3.408	0.719	0.590^**^	0.555^**^	0.536^**^	1

*N* = 535; DP, deliberate practice; TP, task persistence; PC, perceived challenge; CP, creative performance. r: correlation coefficient. ^**^p < 0.01.

### 4.3 The mediation effect test between task persistence and perceived challenge

**Figure 2:**
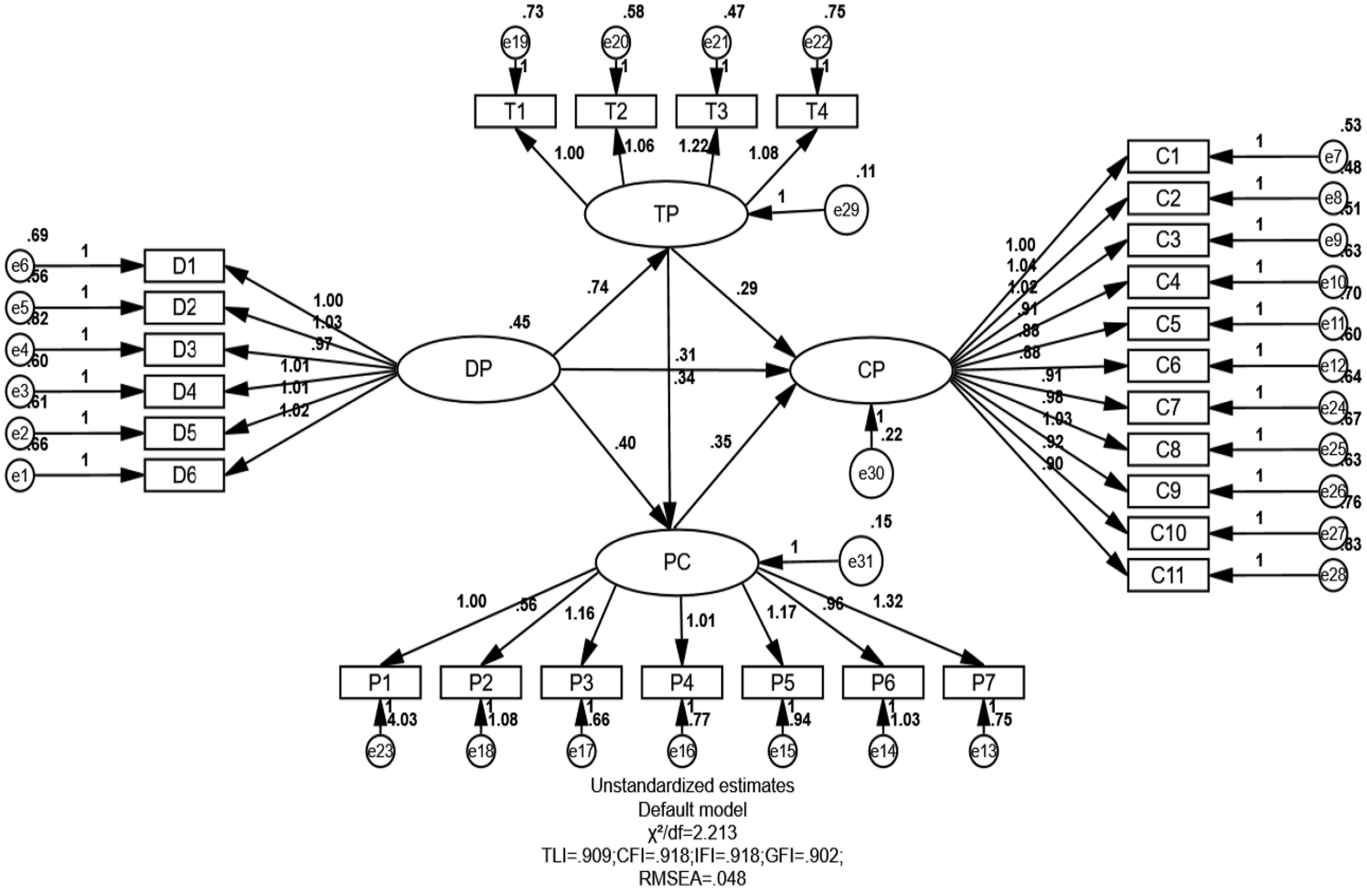
Chain mediation model. DP, deliberate practice; TP, task persistence; PC, perceived challenge; CP, creative performance.

The goodness-of-fit indices for the chained mediation model were assessed using AMOS 24 software, revealing a good model fit, with χ^2^/df = 2.213, NFI = 0.960, GFI = 0.902, RMSEA = 0.048, CFI = 0.918, and TLI = 0.909. This suggests that the hypothesized mediation model adequately represented the observed data. To evaluate the significance of the chained mediation effects, bootstrapping procedures with 5,000 resamples were conducted within AMOS 24. This nonparametric resampling method allowed for the estimation of indirect effects and their 95% bias-corrected confidence intervals, providing strong evidence for mediation without relying on normal distribution assumptions. Indirect effects were deemed statistically significant if the confidence intervals did not include zero. The model fit indices and detailed mediation results for the chained mediation model are illustrated in Figure 2.

First, path analyses of the mediating variables were conducted. As shown in Table 3, the direct path from deliberate practice to creative performance was significant (*β* = 0.309, t = 2.780, *p* < 0.01), supporting Hypothesis 1. The direct effect of deliberate practice on task persistence was also significant (*β* = 0.740, t = 9.955, *p* < 0.01). Furthermore, the direct path from deliberate practice to perceived challenge was significant (*β* = 0.395, t = 3.258, *p* < 0.01). Additionally, the direct path from task persistence to creative performance was statistically significant (*β* = 0.285, t = 2.273, *p* < 0.01). The effect of perceived challenge on creative performance was significant (*β* = 0.351, t = 3.301, *p* < 0.01), as was the direct effect of task persistence on perceived challenge (*β* = 0.338, t = 2.601, *p* < 0.01).

**Table 3:** Analysis of regression relationship among variables.

Item	Effect	SE	*t*	*p*	LLCI	ULCI
DP → CP	0.309	0.043	2.780	0.000	0.214	0.381
DP → TP	0.740	0.034	9.955	0.000	0.582	0.716
DP → PC	0.395	0.044	3.258	0.000	0.256	0.428
TP → CP	0.285	0.041	2.273	0.000	0.133	0.294
PC → CP	0.351	0.040	3.301	0.000	0.171	0.328
TP → PC	0.338	0.043	2.601	0.000	0.194	0.362

*N* = 535; DP, deliberate practice; TP, task persistence; PC, perceived challenge; CP, creative performance.

Using the bias-corrected percentile bootstrap method with 5,000 resamples, the mediating effects of task persistence and perceived challenge in the relationship between deliberate practice and creative performance were examined. The results, summarized in Table 4, indicated that the indirect effect of DP on CP through task persistence yielded a 95% confidence interval ranging from 0.065 to 0.219, with a point estimate of 0.138, demonstrating that task persistence significantly mediates this relationship, supporting Hypothesis 2. Conversely, the indirect effect via perceived challenge showed a CI of 0.045 to 0.135 with an effect size of 0.085, confirming that perceived challenge also serves as a significant mediator (supporting Hypothesis 3). Furthermore, the serial mediation pathway—DP → TP → PC → CP—produced a CI of 0.021 to 0.076, with an indirect effect size of 0.045. All confidence intervals excluded zero, indicating that the mediating effects were statistically significant and thus support Hypothesis 4.

**Table 4:** Mediating effect and effect size.

Path	Effect	The proportion of mediations in the total effect	95% Confidence interval	
			Lower limit	Upper limit
DP → TP → CP	0.138	0.138/0.269 = 51.56%	0.065	0.219
DP → PC → CP	0.085	0.085/0.269 = 31.72%	0.045	0.135
DP → TP → PC → CP	0.045	0.045/0.269 = 16.72%	0.021	0.076

*N* = 535; DP, deliberate practice; TP, task persistence; PC, perceived challenge; CP, creative performance.

Collectively, these findings provide robust empirical support for the mediating roles of both TP and PC. Notably, TP emerged as the stronger mediator, both in the direct path and within the sequential mediation chain. These results highlight the multifaceted mechanisms through which DP enhances creative performance, emphasizing the critical contributions of sustained effort and cognitive engagement in challenging tasks.

## 5 Discussion

### 5.1 The relationship between deliberate practice and creative performance

This study identified a significant positive relationship between deliberate practice (DP) and creative performance (CP). Previous research has demonstrated that DP enhances technical skills and cognitive regulation (Ericsson and Harwell, 2019; Oppici et al., 2021), but the current findings extend this understanding by empirically showing that structured practice contributes not only to performance efficiency but also to creative expression in physical education contexts.

Importantly, this study advances the field by situating creativity within systematic training processes, instead of treating it as a spontaneous or personality-driven trait. The findings suggest that creativity in PE may emerge from repeated cycles of goal-directed effort, feedback integration, and cognitive restructuring (Santos et al., 2016). Thus, rather than viewing deliberate practice and creativity as potentially competing constructs (structure versus freedom), this study demonstrates their compatibility within educational settings.

By empirically linking DP to CP in secondary school PE classes, this research provides evidence that structured, feedback-oriented practice can serve as a developmental pathway for creativity, thereby broadening the conceptualization of creative performance in PE.

### 5.2 The mediating role of task persistence

The mediating role of task persistence (TP) clarifies an important motivational mechanism underlying the DP–CP relationship. While persistence has been widely associated with long-term goal attainment (Rudd et al., 2020; Orth et al., 2017), this study contributes to the discourse by demonstrating that persistence functions not merely as a behavioral outcome of practice but as a psychological bridge that translates structured effort into creative output.

The findings specifically demonstrate that deliberate practice improves students’ sustained engagement, thereby promoting creative performance. This finding deepens our understanding by suggesting that creativity in PE may depend less on momentary inspiration and more on the ability to maintain effort through ambiguity, iterative failure, and refinement.

Furthermore, by empirically modeling TP as a mediator rather than a direct predictor, this study provides a more process-oriented explanation of creativity development. It underscores that motivational endurance is not peripheral but central to the creative learning process in physically demanding contexts.

### 5.3 The mediating role of perceived challenge

The findings also confirm the mediating role of perceived challenge (PC), offering further insight into the cognitive appraisal processes involved in the development of creativity. While previous research has emphasized the motivational value of optimally challenging tasks (Colquitt et al., 2000; Csikszentmihalyi et al., 2018; Pesce et al., 2025), this study advances the literature by empirically positioning perceived challenge as a mechanism through which structured practice influences creative outcomes.

Rather than viewing challenge merely as a contextual variable, the results suggest that students’ subjective interpretation of task difficulty plays a crucial role in determining whether effort transforms into innovation. When students perceive challenges as attainable, they are more willing to experiment with alternative movement strategies and explore novel solutions.

This finding refines existing theoretical assumptions by showing that the effectiveness of deliberate practice not only depends on task design but also on learners’ cognitive appraisal of those tasks. By doing so, the study integrates motivational and cognitive perspectives into a unified explanatory model of creativity in PE.

### 5.4 The chain mediation of task persistence and perceived challenge

A significant finding of this study is the identification of a notable chain mediation pathway involving task persistence and perceived challenge. This pathway systematically explains the mechanisms by which DP promotes creativity. Within structured practice environments, persistence enhances emotional regulation and sustained task engagement, enabling learners to build resilience in the face of difficulties. This foundation, in turn, enhances their cognitive appraisal of task challenges, which ultimately contributes to the emergence of creative behaviors (Santos et al., 2016).

The role of perceived challenge as a cognitively mediated outcome of persistence aligns with ecological dynamics theory, which considers creativity to be a complex result of dynamic interactions among practice behavior, task appraisal, and adaptive response strategies (Seifert and Davids, 2017). Recent research highlights the importance of the “persistence–challenge–innovation” sequence, particularly developing tactical and creative skills in sports (Rudd et al., 2020; Orth et al., 2017). This study supports this model, demonstrating that DP enhances TP, which facilitates positive challenge appraisals that ultimately promote creative performance.

Overall, this research deepens the understanding of creativity in physical education by shifting the focus from isolated predictors to an integrated developmental mechanism. It provides empirical evidence that structured practice, sustained motivation, and adaptive cognitive appraisal collectively form the psychological foundation for creative performance among secondary school students.

### 5.5 Applicability across educational and cultural contexts

Although this study was conducted among Chinese secondary school students, the proposed mechanism linking deliberate practice, task persistence, perceived challenge, and creative performance may have broader relevance across educational systems.

Different societies vary in their pedagogical traditions, curriculum structures, and cultural attitudes toward effort and achievement. In some Western educational contexts, creativity is often emphasized through autonomy and exploration, while in many East Asian settings, structured effort and disciplined practice are more strongly valued. The present findings suggest that these approaches need not be contradictory. Instead, structured practice and creativity can coexist when supported by persistence and positive challenge appraisal.

The chain mediation model identified in this study may therefore be adaptable to other countries, provided that instructional design considers local cultural norms and school environments. In educational systems that prioritize autonomy, structured deliberate practice may offer necessary scaffolding to channel creativity productively. Conversely, in systems emphasizing discipline and repetition, incorporating calibrated challenges and strategies to build persistence may help transform routine practice into opportunities for creative development.

Nevertheless, contextual factors such as class size, assessment systems, teacher training, and societal attitudes toward PE may influence implementation. Future cross-cultural comparative research is needed to test the robustness of this mechanism in diverse social and educational settings.

## 6 Practical significance

The findings of this study have important implications for educational practice, especially in PE. First, the established relationship between DP and CP suggests that instructional design should focus on structured, goal-oriented practice activities that include opportunities for reflection and feedback. Educators should move beyond rote repetition and incorporate adaptive, exploratory elements that foster creativity.

Second, the mediating role of task persistence highlights the need to cultivate students’ motivational endurance. PE programs should integrate incremental goal systems and reinforcement strategies to help students sustain their efforts over time. Enhancing persistence not only supports skill acquisition but also provides the groundwork for innovative problem-solving and decision-making in sports.

Third, the significance of perceived challenge suggests that moderate difficulty and perceived attainability are critical motivational drivers. Educators should design appropriately challenging learning tasks, offering scaffolding and peer collaboration to support learners’ perceptions of competence. This approach will likely increase the depth of student engagement with tasks and encourage creative responses.

Finally, the chain mediation pathway revealed in this study emphasizes the necessity for an integrated pedagogical approach. Rather than concentrating solely on isolated cognitive or motivational strategies, educators should strive to create environments that simultaneously support deliberate engagement, emotional resilience, and constructive perceptions of challenge—conditions vital for sustained creative development.

## 7 Limitations and prospects

While this study makes several theoretical and empirical contributions, it also has limitations that warrant consideration. First, the sample was restricted to secondary school students from a specific regional context, which may limit the generalizability of the findings. Future studies should explore more diverse and cross-cultural populations to validate the model across various educational systems and cultural backgrounds.

Second, the study utilized a cross-sectional design, limiting the ability to infer causal relationships among the variables. Longitudinal or experimental studies would provide stronger evidence for the directionality and temporal dynamics of the proposed model, particularly in tracking the developmental trajectories of creative performance.

Third, reliance on self-reported questionnaires may introduce social desirability bias or subjective inaccuracies. Future research should incorporate behavioral observations, teacher assessments, and peer evaluations to triangulate the data and enhance measurement validity.

Additionally, while the present model is informative, it could be expanded to include other psychological constructs such as intrinsic motivation, self-efficacy, metacognitive awareness, or emotional engagement. Investigating the interactions among these variables could provide a more comprehensive understanding of the psychological foundations of creative behavior in PE.

## 8 Conclusion

This study contributes to the understanding of creativity development in PE by demonstrating a chain mediation mechanism linking deliberate practice, task persistence, perceived challenge, and creative performance among secondary school students. The findings indicate that creativity in PE emerges not merely from spontaneous expression, but from structured effort, sustained engagement, and adaptive cognitive appraisal.

By integrating motivational and cognitive constructs within a single explanatory framework, this research advances theoretical understanding of how creative performance can be systematically cultivated in school-based physical education. Furthermore, the results provide actionable guidance for instructional design, emphasizing the importance of structured practice, persistence cultivation, and optimal challenge calibration.

With appropriate contextual adaptation, the proposed mechanism may offer valuable insights for educational systems beyond China, supporting the global effort to balance skill acquisition and creativity development in physical education curricula.

## Data Availability

The original contributions presented in the study are included in the article/supplementary material, further inquiries can be directed to the corresponding author.
